# The Exploration of Fetal Growth Restriction Based on Metabolomics: A Systematic Review

**DOI:** 10.3390/metabo12090860

**Published:** 2022-09-13

**Authors:** Mengxin Yao, Zhuoqiao Yang, Xin Rong, Xuan Hu, Na Yao, Manting Zhu, Xinnan Wang, Xiaoyan Zhu, Jieyun Yin

**Affiliations:** 1 Department of Epidemiology and Health Statistics, Medical College of Soochow University, Suzhou 215000, China; 2 Suzhou Center for Disease Prevention and Control, Suzhou 215000, China; 3 Institute of Suzhou Biobank, Suzhou 215000, China; 4 School of Public Health, Tongji Medical College, Huazhong University of Science and Technology, Wuhan 430030, China

**Keywords:** fetal growth restriction, metabolomics, biomarker

## Abstract

Fetal growth restriction (FGR) is a common complication of pregnancy and a significant cause of neonatal morbidity and mortality. The adverse effects of FGR can last throughout the entire lifespan and increase the risks of various diseases in adulthood. However, the etiology and pathogenesis of FGR remain unclear. This study comprehensively reviewed metabolomics studies related with FGR in pregnancy to identify potential metabolic biomarkers and pathways. Relevant articles were searched through two online databases (PubMed and Web of Science) from January 2000 to July 2022. The reported metabolites were systematically compared. Pathway analysis was conducted through the online MetaboAnalyst 5.0 software. For humans, a total of 10 neonatal and 14 maternal studies were included in this review. Several amino acids, such as alanine, valine, and isoleucine, were high frequency metabolites in both neonatal and maternal studies. Meanwhile, several pathways were suggested to be involved in the development of FGR, such as arginine biosynthesis, arginine, and proline metabolism, glyoxylate and dicarboxylate metabolism, and alanine, aspartate, and glutamate metabolism. In addition, we also included 8 animal model studies, in which three frequently reported metabolites (glutamine, phenylalanine, and proline) were also present in human studies. In general, this study summarized several metabolites and metabolic pathways which may help us to better understand the underlying metabolic mechanisms of FGR.

## 1. Introduction

Fetal growth restriction (FGR) is an obstetric complication defined as the failure of a fetus to attain its pre-determined intrauterine growth potential, which is also referred to intrauterine growth restriction (IUGR) [1]. Usually, these two terms (FGR and IUGR) are considered to be synonymous and interchangeable [2]. When a newborn’s birth weight falls below a predefined threshold for its gestational age, it is considered as small for gestational age (SGA). Compared with FGR, SGA is an auxological but not an etiological definition. It is often assumed that FGR results in SGA [3].Therefore, we do not distinguish the above-mentioned terms as we aimed to discuss their etiology, and FGR is used below in this article. 

Usually, FGR is classified into early-onset FGR (<32 weeks) and late-onset FGR (≥32 weeks) based on gestational age of diagnosis [4]. With an incidence of approximately 10%, FGR is second only to preterm birth as a cause of infant morbidity and mortality [5]. Not limited to the neonatal period, the adverse effects of poor fetal growth may also last throughout the entire lifespan [6]. For example, several studies have shown that FGR increases the risks of cognitive delay, obesity, cardiovascular disease, and type 2 diabetes in later life [7,8,9,10]. Currently, the main clinical diagnostic methods of FGR are symphysis-fundal height and ultrasound. The former is widely used due to its low cost and convenience [11]. However, maternal obesity, uterine leiomyomas, and polyhydramnios may limit its effectiveness in detecting FGR. Regarding ultrasound, on one hand, its relatively high cost may impede its usage in low resource areas. On the other hand, body habitus, operator experience, and fetal position may influence its accuracy [4]. Meanwhile, the phenotypes of late-onset FGR differ significantly from that of early-onset FGR. Thus, a single fetal biometric measurement is insufficient to evaluate fetal growth, except perhaps in the case of extremely small fetal size [12]. Perhaps, complementary biological tests might help us to identify FGR. In addition, despite FGR is known to be a multifactorial disease that affected by maternal, placental, fetal, and genetic factors [13,14], its etiology and pathogenesis remain unclear. Therefore, enhanced research on the etiology, pathogenesis, and metabolic drivers of FGR is urgently required for accurately monitoring fetal growth.

Metabolomics is an emerging high-throughput technique that enables the comprehensive and systematic identification and quantification of small molecules in biological systems. Previous studies have shown that metabolomics improves our understanding of the pathology of many diseases and is a promising tool for disease diagnosis [15,16]. Therefore, metabolomics may provide us with new insights into FGR.

In this study, we reviewed all metabolomics studies related with FGR in pregnancy over the last 20 years. We also comprehensively summarized and analyzed the information from these studies. The review aimed to (1) better understand the specific metabolic mechanisms of FGR, and (2) identify metabolic biomarkers that can improve diagnostic and predictive capabilities of FGR.

## 2. Materials and Methods

### 2.1. Literature Search

This systematic review was conducted in compliance with PRISMA guidelines [17], and a review protocol was entered into the Prospero database (registration number 356869). We obtained relevant publications from the PubMed and Web of Science databases from January 2000 to July 2022. The search terms were (“metabolome” or “metabolomics” or “metabolite” or “metabonomics” or “metabolic profiling” or “metabolic signature” or “metabolic biomarker” or “metabolic profile” or “metabolic portraits”) AND (“fetal growth restriction” or “intrauterine growth restriction” or “small for gestational age”). Two researchers searched the articles independently, and a third researcher made a final decision in cases of disagreement.

### 2.2. Inclusion and Exclusion Criteria

The inclusion criteria were (1) metabolomics studies for pregnancies, (2) full text in English, and (3) studies recorded the positive or negative relationship between metabolite markers and FGR.

The exclusion criteria were as follows: (1) review articles, (2) non-perinatal studies or FGR fetus disturbed by diet, (3) studies evaluating drug effects, (4) abstracts without full articles, and (5) studies only reported insignificant metabolites.

### 2.3. Data Extraction

**Figure 1:**
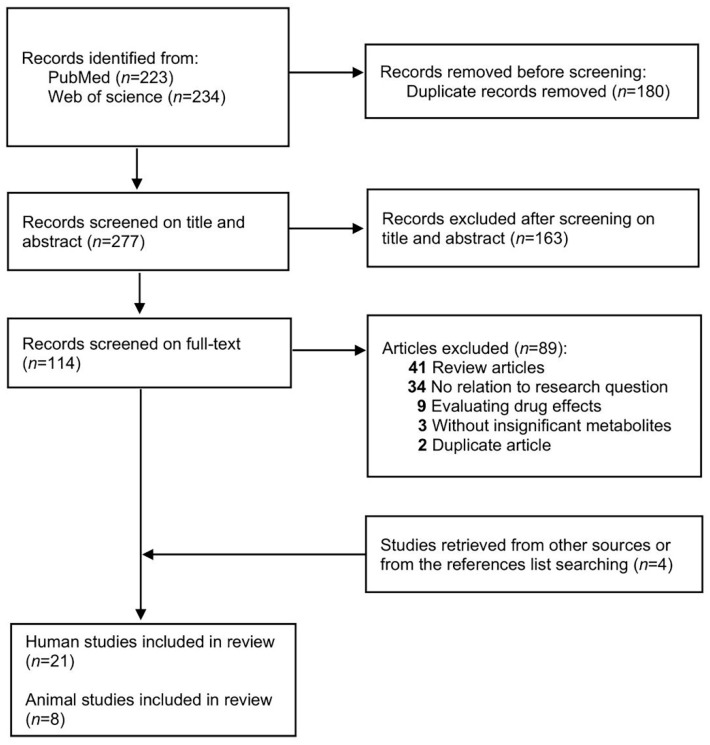
PRISMA flowchart of study selection.

A total of 29 studies, consisting of 21 human studies and 8 animal studies, were eligible for this systematic review (Figure 1). We extracted the following information after reading the full articles and supplementary materials: (1) basic information of included studies, including first author, published year and journal; (2) basic information of subjects, including species, sample sizes, and singleton/twins; (3) study design, diagnostic criteria, or model of FGR, biological specimen, sampling time, and analytic platform; (4) the significant metabolites with changing trends. In addition, studies by the same first or corresponding author were checked as to whether there were overlaps in the content.

### 2.4. Statistical Analysis

The frequencies on biological specimens, targeted/untargeted, analytic platforms, sample sizes, and repeatedly reported biomarkers were computed and charted. Pathway analysis, including enrichment analysis and topology analysis, was performed using the online MetaboAnalyst software (version 5.0, Mcgill University, Montreal, QC, Canada; https://www.metaboanalyst.ca, accessed on 10 July 2022) [18]. The significant pathways were selected based on the criteria of the false discovery rate (FDR) < 0.05.

## 3. Results

### 3.1. Human Neonatal Research

#### 3.1.1. Study Characteristics of Human Neonatal Research

**Figure 2:**
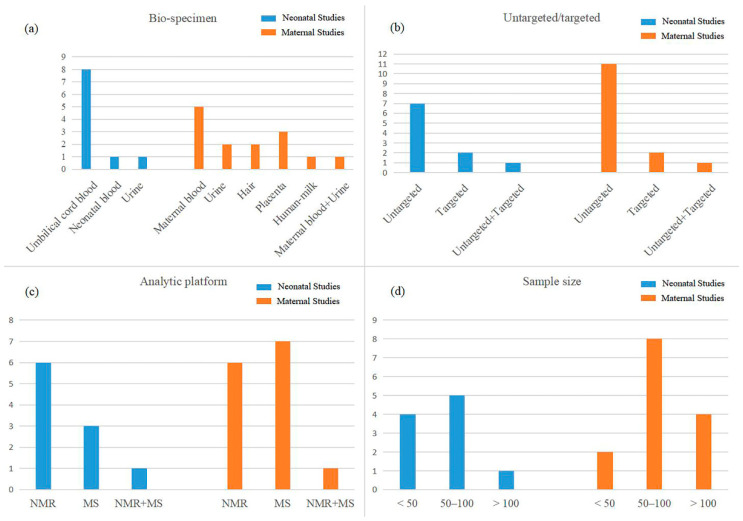
Numbers of the human neonatal and maternal studies according to (**a**) bio-specimen, (**b**) untargeted/targeted, (**c**) analytical platform, and (**d**) sample size.

As shown in Table 1, a total of ten studies [19,20,21,22,23,24,25,26,27,28] on human neonatal samples were included. Eight studies used umbilical cord blood, and the remaining two studies collected neonatal blood and urine (Figure 2a). Seven of the nine studies were untargeted, two were targeted, and the remaining one used untargeted and targeted method simultaneously (Figure 2b). Besides, six metabolomics studies used nuclear magnetic resonance (NMR), three used mass spectrometry (MS), and one study used both (Figure 2c). For the sample sizes, the majority of the studies ranged from 50 to 100 (Figure 2d).

#### 3.1.2. Analysis of High Frequency Biomarkers in Human Neonatal Research

Among the ten studies included, the frequencies of significant metabolites in different studies were counted (Table 2). We found several high frequency biomarkers (reported in ≥3 studies). Ranked by reported frequency, the top nine metabolites were alanine, proline, valine, phenylalanine, glutamine, isoleucine, creatine, tryptophan, and choline. Most high frequency metabolites belong to amino acids. Choline showed a consistent changing trend in FGR, and Sanz-Cortés et al. [24] reported that this biomarker was down-regulated in both early-onset and late-onset FGR.

**Table 2:** High Frequency Metabolic Biomarkers of Human Neonatal Studies and Maternal Studies.

**Neonatal Studies**						
**No**	**Metabolites**	**Total Hits**	**Up**		**Down**	
			**Hits**	**Bio-Specimen**	**Hits**	**Bio-Specimen**
1	Alanine	5	3	Cord serum [19] Dried blood [26] Cord plasma [28]	2	Cord serum [24] (l) Cord plasma [25]
2	Proline	4	3	Cord serum [21] Dried blood [26] Cord plasma [28]	1	Cord plasma [25]
3	Valine	4	2	Cord serum [19,21]	2	Cord serum [24] (l) Dried blood [26]
4	Phenylalanine	4	2	Cord serum [21] Cord plasma [25]	2	Cord serum [19,24] (e)
5	Glutamine	4	2	Cord serum [24] (e) Cord plasma [28]	2	Cord serum [24] (l) Cord plasma [25]
6	Isoleucine	3	2	Cord serum [19,21]	1	Cord plasma [20]
7	Creatine	3	2	Cord serum [24] (e) Urine [27]	1	Cord serum [22]
8	Tryptophan	3	1	Cord serum [21]	2	Cord serum [19] Cord plasma [28]
9	Choline	3	-	-	3	Cord serum [22,24] (e l) Cord plasma [25]
**Maternal Studies**						
**No**	**Metabolites**	**Total Hits**	**Up**		**Down**	
			**Hits**	**Bio-Specimen**	**Hits**	**Bio-Specimen**
1	Alanine	5	1	Maternal serum [19]	4	Urine [29] Maternal hair [30] Maternal plasma [23] Placenta [31]
2	Citrate	4	2	Placenta [31] Human-milk [32]	2	Urine [29] Maternal plasma [23]
3	Valine	4	1	Maternal serum [19]	3	Maternal hair [30] Placenta [31] Human-milk [32]
4	Glycine	4	-	-	4	Urine [29] Maternal hair [30] Placenta [28,31]
5	Isoleucine	3	1	Maternal serum [19]	2	Maternal hair [30] Human-milk [32]
6	Lactate	3	1	Placenta [33]	2	Urine [29] Maternal hair [30]
7	Tyrosine	3	-	-	3	Urine [29] Maternal hair [30] Placenta [28]
8	Aspartate	3	-	-	3	Maternal hair [30] Placenta [28,31]
9	3-Hydroxybutyrate	3	-	-	3	Maternal serum [19,34] Placenta [31]

l, late-onset FGR; e, early-onset FGR.

#### 3.1.3. Metabolic Pathway Analysis of Potential Biomarkers in Human Neonatal Research

**Figure 3:**
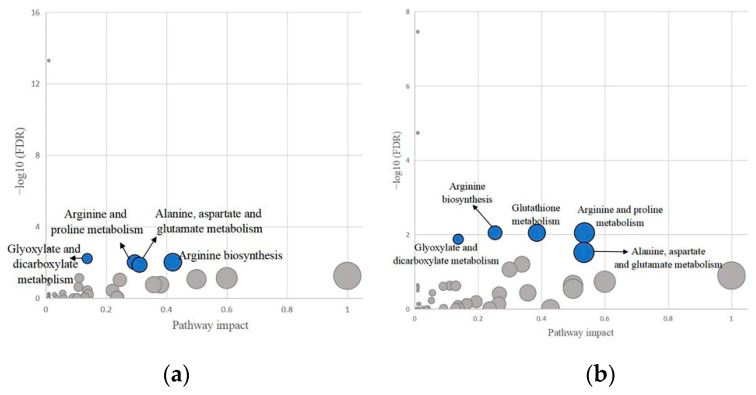
Overview of pathway analysis of human (**a**) neonatal and (**b**) maternal studies. The x-axis is the pathway impact value calculated based on topology analysis, while the y-axis indicates the significance level in enrichment analysis. Each circle in the figure represents a metabolic pathway. The circle size reflects the pathway impact value. And the blue circle indicates the pathway is significantly enriched (FDR < 0.05) and has relatively high impact value.

To understand the metabolic pathways that these significant biomarkers are involved in, we imported all the reported metabolites in neonatal research into the online MetaboAnalyst 5.0 software for pathway analysis. The results showed that six pathways were significantly enriched (FDR < 0.05, shown in Table S1). Particularly, four pathways ((1) glyoxylate and dicarboxylate metabolism; (2) arginine biosynthesis; (3) arginine and proline metabolism; (4) alanine, aspartate, and glutamate metabolism) possessed relatively high impact values (Figure 3a).

### 3.2. Human Maternal Research

#### 3.2.1. Study Characteristics of Human Maternal Research

A total of fourteen articles [19,23,28,29,30,31,32,33,34,35,36,37,38,39] of maternal studies were included in the final analysis (Table 3). The characteristics of human maternal and neonatal research were similar. Among these included studies, maternal blood was the most common bio-specimen. Urine, hair, human-milk, and placenta were also used (Figure 2a). Similar to neonatal studies, researchers usually conducted untargeted studies (Figure 2b). For the analytical platform, six studies were NMR, seven were MS, and the remaining one used both (Figure 2c). The number of cases among the maternal studies varied from 10 to 175, while the sample sizes of most studies were between 50 and 100 (Figure 2d).

#### 3.2.2. Analysis of High Frequency Biomarkers in Human Maternal Research

A total of nine high frequency metabolites (reported in ≥ 3 studies) were revealed in maternal derived studies, including alanine, citrate, valine, glycine, isoleucine, lactate, tyrosine, aspartate, and 3-hydroxybutyrate (Table 2). These metabolites belong to amino acids and organic acids. Among them, glycine, tyrosine, aspartate and 3-hydroxybutyrate showed a consistent down-regulation trend in all reported human maternal articles. 

#### 3.2.3. Metabolic Pathway Analysis of Potential Biomarkers in Human Maternal Research

After inputting significant metabolites of maternal research into MetaboAnalyst, a total of seven pathways were identified (FDR < 0.05, Table S2). Five pathways had relatively high impact values, they were (1) glutathione metabolism; (2) arginine biosynthesis; (3) arginine and proline metabolism; (4) glyoxylate and dicarboxylate metabolism; (5) alanine, aspartate, and glutamate metabolism (Figure 3b).

### 3.3. Comparison between Human Neonatal and Maternal Research

A number of high frequency metabolites, especially amino acids (alanine, valine, and isoleucine), were simultaneously revealed from neonatal and maternal research. In addition, four pathways with FDR < 0.5 were significantly enriched in both neonatal and maternal studies. They were glyoxylate and dicarboxylate metabolism; arginine biosynthesis; arginine and proline metabolism; and alanine, aspartate, and glutamate metabolism. 

### 3.4. Classification Potential of the Metabolic Biomarkers

Seven studies [19,22,30,31,34,36,38] have assessed the potential of metabolic biomarkers or biomarkers panels to predict or diagnose FGR (Table 4). Moros et al. [19] calculated the area under the receiver operating curve (AUC) of several metabolites one at a time, resulting in all of the AUC values greater than 0.750. Sovio et al. [36] explored the discriminating potential of various combinations of the four metabolites (1-(1-enyl-stearoyl)-2-oleoyl-GPC (P-18:0/18:1); 1,5-anhydroglucitol; 5α-androstan-3α,17α-diol disulfate; N1,N12-diacetylspermine), yielding AUC values ranging from 0.640 to 0.780. Bahado-Singh and coworkers [22,31] reported that the incorporation of metabolites with maternal factors did not improve predictive accuracy over the best biomarkers panels. Sulek et al. [30] uncovered a panel of five metabolite parameters (lactate, levulinate, 2-methyloctadecanat, tyrosine, and margarate) with an AUC value of 0.998, suggesting that this model’s ability to distinguish FGR was remarkable.

### 3.5. Animal Studies

#### 3.5.1. Study Characteristics of Animal Model Research

According to the inclusion and exclusion criteria, a total of 8 studies [40,41,42,43,44,45,46,47] on animal models were included. The characteristics of these studies are presented in Table 5. The animals involved were rat, sheep, calf, and piglet. These studies were all conducted in newborn animals. Compared with human studies, the types of biological samples in animal studies were diversity, including fetal plasma, umbilical cord plasma, skeletal muscle, muscle, liver, and kidney. Untargeted method was commonly used to detect metabolites, and the analysis platform was always MS in animal studies. Most animal studies had sample size of less than 20. Only one study [47] for fetal rats had 53 cases and 57 controls. 

#### 3.5.2. Analysis of High Frequency Biomarkers in Animal Research

Four high frequency (reported in ≥ 3 studies) differential metabolites extracted from these 8 animal studies are shown in Table 6. These metabolites are arginine, reported four times, and glutamine, phenylalanine, and proline, which occurred three times. These differential metabolites in FGR animal fetus were all amino acids, which was similar to the human study. Among them, glutamine, phenylalanine, and proline were also high frequency metabolites reported in human neonatal studies.

## 4. Discussion

In this systematic review, we comprehensively screened and analyzed metabolomics studies on FGR around delivery. In human research, neonatal and maternal studies each identified several possible metabolic pathways and four of them were consistent, suggesting similar metabolic patterns of mothers and newborns in FGR pregnancies. Three high frequency metabolites (glutamine, phenylalanine, and proline) found in the human neonatal studies were also reported in the animal model studies. In addition, several metabolic biomarkers or biomarker panels showed classification potential for FGR.

### 4.1. Potential Metabolic Dysregulations of FGR

#### 4.1.1. Amino Acid Metabolism Disorder

All of the three high frequency metabolites in both human and animal neonatal studies were amino acids. Glutamine [24,25,28,43,44,47] is the most abundant amino acid in the body [48], which is not only important for energy supply, but is also a precursor for nucleotide synthesis [49]. Phenylalanine [19,21,24,25,43,44,47] belongs to aromatic amino acids, as precursor of the monoamine neurotransmitters, serotonin and catecholamines in the brain [50]. Proline [21,25,26,28,40,43,44] plays a role in maintaining cellular homeostasis and modulating mitochondrial functions [51]. 

In addition, several amino acids were frequently reported to be downregulated in human maternal studies. The reported valine [19,30,31,32] and isoleucine [19,30,32] are essential branched chain amino acids (BCAAs), which can activate mTOR signaling pathway to stimulate protein synthesis and cell growth [52]. Lowered concentrations of BCAAs may result in disturbed transportation of placental amino acid to fetus and therefore impaired fetal growth [53,54]. Alanine [19,23,29,30,31] was reported several times. Alanine can be synthesized from BCAAs. As a key amino acid in nitrogen metabolism, alanine provides energy for muscle tissue and the central nervous systems via gluconeogenic pathway [55]. Glycine showed consistent down-regulated trend in the included studies [28,29,30,31]. It is involved in body’s collagen, immune response, and plays a crucial role as neurotransmitter. 

In general, amino acids are the precursor of many biologically active molecules, and play a key role in regulating cell metabolism, proliferation, differentiation, and growth [56]. Our results supported the view that low concentrations of maternal amino acids play an important role in the occurrence and development of fetal abnormal growth. 

#### 4.1.2. Insulin Deficiency 

Insulin is an important hormone for intrauterine growth. Pathway analysis suggested that pathways of alanine, aspartate, and glutamate metabolism, which may affect insulin secretion, are related to FGR. Alanine [19,23,24,25,26,28,29,30,31,41,45] was the most frequently reported metabolite in the included studies. A previous study suggested alanine metabolism provides key stimulus-secretion coupling factors that are critical for promoting insulin secretion [57]. Glutamate [21,30,34], which was revealed to be an important mediator in the amplification of insulin secretion [58], is the major precursor of the repeatedly reported glutamine [24,25,28,33,34,43,44,47]. Fetal insulin deficiency can not only decrease fetal tissues to uptake and utilize nutrient, but also lower circulating concentrations of insulin-like growth factors (IGFs) [59]. Animal studies showed that maternal plasma IGFs correlate positively with fetal growth and birth weight [60]. In addition to suppressing fetal growth during pregnancy, the effects of insulin deficiency may persist into postpartum and even adulthood. To compensate for intrauterine growth restriction, the neonates usually are more insulin-sensitive and then undergo a period of accelerated postnatal growth [61], which is associated with increased risk of developing insulin resistance and eventually type 2 diabetes [62].

#### 4.1.3. Oxidative Stress

Oxidative stress refers to the imbalance between reactive oxygen species (ROS) and protective antioxidants. Overproduction of ROS can destroy normal placental functions. In the current study, mothers gave birth to FGR infants showed a disorder of glutathione metabolism. Glutathione, a natural body antioxidant, is a tripeptide composed of glycine, cysteine, and glutamate. Disturbance of glutathione metabolism may result in elevated oxidative products and increased oxidative stress [63], which could induce placental vascular lesions and lead to fetal compromise [9].

#### 4.1.4. NO Synthesis Dysfunction

Nitric oxide (NO) is the main vasodilatory agent of placenta and is involved in implantation, fetoplacental vascular reactivity, and placental perfusion [64]. This gas is produced from arginine [22,28,40,41,43,47] through activation of nitric oxide synthases [65]. Arginine and proline metabolism and arginine biosynthesis were identified by pathway analysis, supporting NO synthesis dysfunction in FGR.

#### 4.1.5. Energy Metabolism Disorder

The growth of the placenta and fetus during pregnancy increases the demand for energy [66]. The main source of cellular energy is the tricarboxylic acid cycle (TCA cycle) [67]. Glyoxylate and dicarboxylate metabolism can regulate TCA cycle via metabolites [68], such as the reported citrate [23,27,29,31,32,41] and glycine [28,29,30,31]. The identified glyoxylate and dicarboxylate metabolism pathway suggested disruptions in energy metabolism in FGR.

### 4.2. Reliability of Metabolic Biomarkers/Metabolite Panels

We identified some frequently reported metabolites, which strengthened our confidence in the exploration for metabolic biomarkers of FGR. Unfortunately, the change trends of some metabolites were not consistent across studies. Some of the inconsistencies may be explained by differences in biological samples, study populations, subject characteristics, analysis platforms, and other aspects. Therefore, more studies are needed to identify the metabolites involved in FGR.

Several metabolomics studies used single metabolites [19,34] or panels [22,30,31,36,38] to predict or diagnose FGR, showing decent discriminative ability. However, the application of these biomarkers in clinical practice is still insufficient. Further studies, such as cohorts with large sample sizes, are needed to obtain reliable conclusions. In addition, the combination of metabolic biomarkers with traditional predictive biomarkers or clinical parameters also needs to be explored to obtain more effective clinical models.

### 4.3. Limitations of Current Metabolomics Studies on FGR

Several limitations should be addressed. First, differences in study designs, diagnostic criteria of FGR, metabolomic testing platforms, and statistic methods may lead to inconsistent results, creating challenges in identifying potential metabolic biomarkers. For example, due to FGR or IUGR usually corresponding with SGA, studies on SGA were also included. Pathological differences between them may influence the results. Second, the majority of studies had a relatively small sample size, which may limit the statistical power and reliability of their results. In addition, many studies did not have validations. Before translating the results into clinical practice, multiple independent validations, including in vitro and in vivo studies, are necessary. Third, profiling of metabolites may be greatly dynamic and influenced by diet, immune status, lifestyle, and environmental factors [69]. Therefore, strict quality control is necessary for metabolomics studies. Finally, metabolomics only provides insights into the mechanisms of FGR, and it is necessary to combine other omics (e.g., genomics, transcriptomics, and proteomics) to make deeper and more comprehensive exploration of FGR.

## 5. Conclusions

In summary, this study presents a systematic review and analysis of metabolomics research. We identified a series of small-molecule metabolites, mainly amino acids, that were altered in FGR. The reported metabolic biomarkers and pathways suggest underlying metabolic mechanisms in the development and progression of FGR. However, current metabolomics studies on FGR are still in the preliminary development stage and more comprehensive metabolomic studies should be encouraged.

## Figures and Tables

**Table 1:** Characteristics of the Human Neonatal Studies.

Ref	Case Sample Size	Control Sample Size	Diagnostic Criteria	Bio-Specimen	Sampling Time	Analytic Platform	Targeted/ Untargeted	Upregulated	Downregulated
Georgios Moros (2021) [19]	41	36	BW < 10th customized centile and abnormal umbilical artery RI	Umbilical cord serum	Not mentioned	^1^H NMR	Untargeted	Alanine; Leucine; Isoleucine; Valine	Phenylalanine; Glycerol; Tryptophan
Lina Youssef (2021) [20]	43	86	EFW and BW < 10th centile and abnormal cerebroplacental ratio or abnormal uterine artery pulsatility index, or BW < 3rd centile	Umbilical cord plasma	Immediately after delivery	^1^H NMR	Untargeted	Triglycerides (IDL); Cholesterol (IDL)	Cholesterol (HDL); Isoleucine
Donata Favretto (2012) [21]	22	21	EFW < 10th percentile	Umbilical cord serum	Immediately after delivery	LC-HRMS	Untargeted	Valine; Isoleucine; Glutamate; Methionine; Dopamine; Histidine; Proline; Phenylalanine; Uric acid; Caffeine; 5-Methyl-2-undecenoic acid; Tryptophan; Kynurenine; Leu pro; Thyronine; Oleic acid; Hexadecanedioic acid; Phe phe; Arg cys asn; Arg phe arg; Trp arg arg; 1-Hydroxyvitamin D_3_ 3-dglucopyranoside	None
Ray Oliver Bahado-Singh (2019) [22]	39	39	BW < 10th percentile	Umbilical cord serum	Within 20 min of delivery	LC-MS/MS ^1^H NMR	Untargeted	Lysine; Threonine; Dopa; Kynurenine; LysoPC a C18:1; LysoPC a C20:3; PC aa C36:1; PC aa C36:3; PC aa C38:3; PC ae C44:5; Arginine	Creatinine; C0; C10:1; C12:1; C16:1; C2; C4; C4-OH; PC aa C24.0; PC aa C26.0; PC aa C28:1; PC aa C30:0; PC aa C32:0; PC aa C32:1; PC aa C34:2; PC aa C36:4; PC aa C36:6; PC aa C38:4; PC aa C38:5; PC aa C40:4; PC aa C40:6; PC aa C42:2; PC aa C42:4; PC aa C42:5; PC aa C42:6; PC ae C30:2; PC ae C36:0; PC ae C36:3; PC ae C36:5; PC ae C38:4; PC ae C38:6; PC ae C42:1; 2-Hydroxybutyrate; 3-Hydroxybutyrate; Acetate; Acetoacetate; Choline; Creatine; Formate
Jezid Miranda (2018) [23]	27	28	BW < 3rd centile and/or abnormal uterine artery doppler and/or abnormal cerebroplacental ratio	Umbilical cord plasma	Immediately after delivery	^1^H NMR	Untargeted +targeted	Cholesterol (VLDL); Triglycerides (VLDL); Triglycerides (IDL); Acetate; Formate	None
Magdalena Sanz-Cortés (2013) [24]	20	23	BW < 10th centile and abnormal umbilical artery	Umbilical cord serum	At delivery	^1^H NMR	Untargeted	Triglycerides; Creatine; Glutamine (Early-onset FGR)	Glucose; Choline; Phenylalanine (Early-onset FGR) Choline; Glutamine; Tyrosine; Valine; Leucine; Alanine (Late-onset FGR)
Carmen Ivorra (2012) [25]	20	30	BW < 10th percentile	Umbilical cord plasma	Immediately after delivery	NMR	Untargeted	Citrulline; Phenylalanine	Proline; Choline; Glutamine; Alanine; Glucose
Aviv Schupper (2021) [26]	6380	61,068	BW < 10th percentile	Neonatal dried blood	Between 36 and 72 h from birth	HPLC-MS	Targeted	Alanine; Methionine; Proline; Carnitine	Valine
Angelica Dessı` (2014) [27]	12	17	Ultrasonographically in the prenatal period and BW < 10th percentile	Neonatal urine	Within 8 h of delivery	^1^H NMR	Untargeted	Citrate; Creatinine; Creatine; Myo-inositol; Betaine/Trimethylamine N-oxide; Glycine	None
Juan Manuel Chao de la Barca (2022) [28]	15	15	BW < 10th percentile and doppler abnormalities	Umbilical cord plasma	Not mentioned	LC-MS/MS FIA-MS/MS	Targeted	C0; C4; C2; Alanine; Asparagine; Tyrosine; Glutamine; Proline; Alpha-aminoadipic acid; Trans-4-hydroxyproline; Spermine; LysoPC a C26:1; PC aa C32:0; PC aa C24:0	LysoPC a C18:0; LysoPC a C17:0; LysoPC a C18:1; LysoPC a C20:3; LysoPC a C18:2; LysoPC a C20:4; LysoPC a C16:1; LysoPC a C16:0; PC ae C40:6; PC ae C42:5; PC aa C38:6; PC aa C36:0; PC aa C42:0; PC aa C42:6; PC ae C42:4; PC ae C40:2; PC ae C38:6; PC aa C36:1; PC ae C40:3; PC ae C40:5; PC ae C40:4; PC aa C36:3; PC ae C42:2; PC ae C40:1; PC aa C40:6; PC ae C42:3; PC ae C44:5; PC aa C38:0; PC ae C38:0; PC aa C36:6; PC aa C38:3; PC ae C44:4; PC ae C38:3; PC ae C44:6; PC ae C36:3; SM(OH)C22:1; SM(OH)C24:1; SM C24:0; SM C26:0; Tryptophan

BW, birth weight; RI, resistance indices; EFW, estimated fetal weight; IDL, intermediate density lipoprotein; HDL, high density lipoprotein; VLDL, very low density lipoprotein.

**Table 3:** Characteristics of the Human Maternal Studies.

Ref	Case Sample Size	Control Sample Size	Diagnostic Criteria	Bio-Specimen	Sampling Time	Analytic Platform	Targeted/ Untargeted	Upregulated	Downregulated
Georgios Moros (2021) [19]	41	36	BW < 10th customized centile and abnormal umbilical artery RI	Maternal serum	During delivery	^1^H NMR	Untargeted	Alanine; Leucine; Isoleucine; Valine	Phenylalanine; Glycerol; 3-Hydroxybutyrate
Chelsea M. Clinton (2020) [35]	30	30	BW < 10th percentile	Urine	10 w	GC-MS	Untargeted	Benzoic acid; Malonic acid; 2-Ketoleucine/ketoisoleucine; 2-Ketobutyric acid; 2-Methylglutaric acid; Acetoacetate	None
					26 w			1,2-Propanediol; Kynurenic acid; N-heptanoic acid; Benzoic acid	None
Léa Maitre (2014) [29]	36	275	BW < 10th percentile of predicted BW distribution	Urine	11–13 w	^1^H NMR	Untargeted	None	Tyrosine; Lactate; Alanine; Acetate; Citrate; Trimethylamine; Glycine; Formate
Ulla Sovio (2020) [36]	175	299	BW < 3rd percentile, or BW between the 3rd and 10th percentile and the lowest decile of fetal abdominal growth velocity	Maternal serum	20/28/36 w	UPLC-MS/MS	Untargeted	1-(1-Enyl-stearoyl)2-oleoyl-GPC (P-18:0/18:1); 1,5-Anhydroglucitol; Cotinine N-oxide; 4-Androsten-3beta,17beta-diol monosulfate; Hydroxycotinine; Acisoga; 3-Hydroxycotinine glucuronide; O-cresol sulfate; Dehydroisoandrosterone sulfate	5Alpha-androstan-3Alpha,17alpha-diol disulfate; Estriol 3-sulfate; 4-Cholesten-3-one; Pregnanolone/allopregnanolone sulfate; 5Alpha-pregnan-3alpha,20beta-diol disulfate 1; N1,N12-diacetylspermine; 17Alpha-hydroxypregnanolone glucuronide; 5Alpha-pregnan-3beta,20beta-diol monosulfate; Progesterone; Pregnanediol-3-glucuronide
Karolina Sulek (2014) [30]	41	42	BW < 10th corrected birth centile	Maternal hair	26–28 w	GC-MS	Untargeted	Palmitate; 2-Methyloctadecanoate; Myristate; Margarate; Stearate; Dodecanoate; Octanoate; Heptadecane; Nicotinamide	3-Hydroxybenzoate; Levulinic acid; 1-Aminocyclopropane-1-carboxylate; Citraconate; Lactate; Glycine; Proline; Isoleucine; Serine; Leucine; Glutamate; Phenylalanine; Alanine; Valine; Aspartate; Threonine; Tyrosine; Methionine; Lysine; Pyroglutamate; Ornithine; Glutathione
Jezid Miranda (2018) [23]	27	28	BW < 3rd centile and/or abnormal uterine artery doppler and/or abnormal cerebroplacental ratio	Maternal plasma	2–4 h after delivery	^1^H NMR	Untargeted + targeted	None	Triglycerides (HDL); Alanine; Citrate; 2-oxoisovaleric acid; Pyruvate
Ray Oliver Bahado-Singh (2020) [31]	19	30	BW < 10th percentile	Placenta	Within 20 min of delivery	DI-LC-MS/MS ^1^H NMR	Untargeted	Citrate	3-Hydroxybutyrate; Glycine; D-Glucose; t4-OH-Pro; Symmetric dimethylarginine; Sarcosine; Kynurenine; Methionine sulfoxide; Alanine; Aspartate; Spermidine; Valine; PC ae C424; Myo-inositol
Despina D. Briana (2020) [32]	19	60	BW ≤ 10th customized percentile	Human-milk	Within 3–4 d of delivery	^1^H NMR	Untargeted	Citrate; Choline; Lactose; N-acetylglutamine; Phosphocoline	Isoleucine; Valine
Thibaut D. J. Delplancke (2018) [37]	20	73	BW < 10th customized centile	Hair	In the second trimester	GC-MS LC-MS	Untargeted	Myristic acid; Pentadecanoic acid; Margaric acid	None
Abdullah Karaer (2022) [33]	10	14	AC/EFW ratio < 3rd percentile	Placenta	Within 5 min of delivery	^1^H HR-MAS NMR	Untargeted	Lactate; Glutamine; Glycerophosphocholine; Phosphocholine; Taurine; Myo-inositol	None
Chaelin Lee (2022) [38]	56	56	BW < 10th percentile	Maternal serum	10–14 w	LC-MS	Targeted	Tetrahydrocortisol	21-Deoxycortisol
Aude-Claire Morillon (2021) [39]	40	40	BW < 10th percentile	Urine	20 w	UPLC-MS	Untargeted	None	Sulfolithocholic acid; Estriol-16-glucuronide; 4-Hydroxybenzaldehyde; Neuromedin N; D-Glucuronic acid; 18-Hydroxycortisol; Beta-1,4-Mannosyl-N-acetylglucosamine
	40	40		Maternal serum				PE(P-31:0); PE(42:1); PE(36:4); PS(O-37:0); PS(41:5); PS(37:2); PS(43:6); PS(P-34:0); PC(O-42:4); PC(40:5); PC(38:6); LysoPC(16:0); PA(O-36:2); LysoPA(18:1); PI(37:1); PI(P-33:1); PGP(38:4); PGP(40:4); PG(36:6); PG(39:8); PG(38:4); DG(44:4); DG(O-34:1); N,N-dimethyl arachidonoyl amine; N-palmitoyl valine; SM(34:1) Ganglioside GA2 (40:1); Cer(34:0); Cer(39:2)	CL(72:2); TG(64:15); 8S-hydroxy-hexadecanoic acid; CE(17:0)
Katie L. Powel (2018) [34]	34	82	EFW < 10th percentile and abnormal placental vascular resistance	Maternal serum	26–41 w	^1^H NMR	Untargeted	Glutamate; Glutamine	3-Hydroxybutyrate
Juan Manuel Chao de la Barca (2022) [28]	20 + 24	20 + 22	BW < 10th percentile and doppler abnormalities	Placenta	Within 30 min after delivery	LC-MS/MS FIA-MS/MS	Targeted	C0; C2; C4; C4-OH; C16; C18; C18:1; C5; C16:1; C18:2; Tryptophan; Creatinine; PC ae C36:5; Hexose	Aspartate; Threonine; Kynurenine; Putrescine; Spermidine; Trans-4-hydroxyproline; LysoPC a C18:0; LysoPC a C20:3; LysoPC a C20:4; PC aa C36:1; PC aa C38:0; PC aa C40:3; PC ae C42:3; SM C24:0 SM C26:0; SM C24:1; SM (OH) C22:1; Glycine; Serine; Arginine; Tyrosine; Alpha-aminoadipic acid; LysoPC a C16; LysoPC a C16:1; LysoPC a C17:0; LysoPC a C18:1; LysoPC a C18:2; PC aa C32:0; PC ae C34:0; PC ae C40:2; PC ae C44:5; SM C26:1; SM(OH) C22:2; SM(OH) C24:1; Methionine; Carnosine; LysoPC a C24:0; LysoPC a C28:1; PC aa C36:3; PC aa C38:3; PC aa C42:0; PC ae C30:0; PC ae C30:2; PC ae C36:1; PC ae C36:2; PC ae C36:3; PC ae C38:2; PC ae C38:3; PC ae C40:3; PC ae C40:4; PC ae C42:4; PC ae C42:6; SM C16:1; SM(OH) C14:1

RI, resistance indices; BW, birth weight; HDL, high density lipoprotein; AC, abdominal circumference; EFW, estimated fetal weight.

**Table 4:** The Potential of Metabolic Markers for the Prediction and Diagnosis of FGR.

Ref	Bio-Specimen	Potential Biomarkers	Sensitivity	Specificity	AUC
Georgios Moros (2021) [19]	Maternal and umbilical cord blood	Alanine	-	-	0.871 (Umbilical cord blood) 0.792 (Maternal blood)
		Isoleucine	-	-	0.795 (Umbilical cord blood) 0.812 (Maternal blood)
		Leucine	-	-	0.816 (Umbilical cord blood) 0.773 (Maternal blood)
		Valine	-	-	0.785 (Umbilical cord blood) 0.786 (Maternal blood)
		Phenylalanine	-	-	0.779 (Umbilical cord blood) 0.75 (Maternal blood)
		Glycerol	-	-	0.853 (Umbilical cord blood) 0.751 (Maternal blood)
		Tryptophan	-	-	0.751 (Umbilical cord blood)
		3-Hydroxybutyrate	-	-	0.774 (Maternal blood)
Ulla Sovio (2020) [36]	Maternal serum	(A)1-(1-Enyl-stearoyl)-2-oleoyl-GPC (P-18:0/18:1)	-	-	0.640
		(B)1,5-Anhydroglucitol	-	-	0.650
		(C)5α-Androstan-3α,17α-diol disulfate	-	-	0.690
		(D)N1,N12-diacetylspermine	-	-	0.660
		A × B	-	-	0.700
		C × D	-	-	0.710
		(A × B)/ (C × D)	-	-	0.780
Karolina Sulek (2014) [30]	Maternal hair	Lactate + levulinate + 2-methyloctadecanate + tyrosine + margarate	-	-	0.998
Ray O. Bahado-Singh (2020) [31]	Placenta	3-Hydroxybutyric acid + glycine + PC aa C420	0.867	0.842	0.912
		LysoPC a C261 + PC ae C382 + PC ae C446	0.867	0.737	0.796
		3-Hydroxybutyric acid + glycine + PC aa C420 + medical disorder + maternal age + prior FGR + race + gravidity	0.833	0.789	0.832
Ray Oliver Bahado-Singh (2019) [22]	Cord blood serum	Creatinine + C14 + C2 + C4 + LysoPC.a.C16:1 + LysoPC.a.C18:1 + LysoPC.a.C20:3 + LysoPC.a.C20:4 + LysoPC.a.C28:1 + PC.aa.C24:0 + PC.aa.C36:4 + PC.aa.C38:4 + PC.aa.C42:4 + EDTAca_N + creatine	0.870	0.830	0.910
		LysoPC.a.C16:1 + C2 + creatinine + LysoPC.a.C18:2 + LysoPC.a.C18:1 + LysoPC.a.C20:3 + PC.aa.C24:0 + C6.C4:1.DC. + C4 + C10:1 + C16:1 + C12:1 + C12 + C0 + LysoPC.a.C28:1	0.830	0.850	0.870
		Creatinine + LysoPC.a.C16:1 + LysoPC.a.C20:3 + C2 + LysoPC.a.C18:2 + C4 + C12:1 + EDTAca_N + C6.C4:1.DC. + taurine + C16:2 + C0 + putrescine + PC.aa.C24:0 + LysoPC.a.C28:1	0.850	0.790	0.880
		Creatinine + C2 + C4 + LysoPC.a.C16:1 + LysoPC.a.C2:.3 + LysoPC.a.C28:1 + PC.aa.C24:0	0.830	0.870	0.880
		Creatinine + C2 + C4 + LysoPC.a.C16:1 + LysoPC.a.C2:.3 + LysoPC.a.C28:1 + PC.aa.C24:0 + prior FGR + maternal age + gravity + race	0.700	0.730	0.690
Chaelin Lee (2022) [38]	Maternal serum	21-DeoxyF + F/21-deoxyF(cortisol to 21-deoxycortisol) + THF/F(tetrahydrocortisol to cortisol)	0.734	0.817	0.824
Katie L. Powel (2018) [34]	Placenta	3-Hydroxybutyrate	0.939 0.364	0.300 0.545	0.623 (Discovery) 0.581 (Validation)

**Table 5:** Characteristics of the Animal Studies.

Ref	Case Sample Size	Control Sample Size	FGR Model	Bio-Specimen	Sampling Time	Analytic Platform	Targeted/ Untargeted	Upregulated	Downregulated
Alexandre-Gouabau (2011) [40]	4	10	Fetal rats of low protein diet maternal rats	Fetal plasma	Postnatal 0 d	LC-HRMS	Untargeted	None	Proline; Arginine; Histidine
Eileen I. Chang (2019) [41]	10	8	Fetal sheep of placental insufficiency	Fetal skeletal muscle	Late gestation	HPLC	Untargeted	2-Oxo-7-methylthioheptanoic acid; 2-Hydroxyglutarate; N-acetylneuraminate	Cystathionine; N-acetylcitrulline; Gamma-glutamylalanine; N-carbamyl-L-glutamate; Gamma-glutamylcysteine; Arginine; Aspartate; N,N-dimethylglycine; Adenosine; 5-Hydroxylysine; 4-Hydroxyproline
				Fetal arterial and venous plasma				4-Pyridoxate; 2-Aminoadipate; Triacanthine; Dopamine; Uric Acid; Citrate; Adrenaline; Carnitine; 5,6-Dihydrothymine; Arabitol; Guanine; Taurine; Dehydroascorbate; Alanine	Orthophosphate; Diphosphate; 3D-(3,5/4)-trihydroxycyclohexane-1,2-dione; Fructose 1,6-bisphosphate; 5-Hydroxylysine; 2-Methyleneglutarate; N-Acyl-D-mannosaminolactone; Pyruvate; 1,4-Beta-D-xylan; Glucose; Cys gly; Ascorbate; Arginine; Sn-glycerol 3-phosphate; Succinate; Ribose
Shimeng Huang (2019) [42]	6	6	Piglets with a birth weight 2 sd below the mean	Fetal plasma	Immediately after birth	UPLC-MS	Untargeted	6’-Sialyllactose; Faradiol; Neotigogenin; Zymosterol intermediate 2, 13, 14-Dihydro PGF-1 alpha; 6, 10, 14-Trimethyl-5, 9, 13-pentadecatrien-2-one	Serotonin; Dihydrowyerone
Gang Lin (2012) [43]	9	9	Piglets with a birth weight 2 sd below the mean	Umbilical vein plasma	90 d of gestation	HPLC-Q-TOFMS	Untargeted	Pyroglutamic acid	Proline; Valine; Phenylalanine; Isoleucine; Leucine; Tyrosine; Glutamine; Arginine; Dodecanoylcarnitine
	9	9			110 d of gestation			Creatinine; Carnitine; Pyroglutamic acid	Tryptophan; Glutamine; Arginine; Dodecanoylcarnitine
Susumu Muroya (2021) [44]	4	4	Calves of maternal undernutrition	Fetal muscle	260 ± 8.3 d of gestation	CE-TOFMS	Untargeted	Carnosine; Glutamine; Glycerol; Creatine; N^6^-methyllysine; Phosphorylcholine; Phenylalanine; Proline	Myo-inositol 2-phosphate; 2-Aminoethylphosphonic acid
Susumu Muroya (2022) [45]	4	4	Calves of maternal undernutrition	Fetal liver	260 ± 8.3 d of gestation	CE-TOFMS	Untargeted	Aspartate; Betaine aldehyde; Glycerol; 3-Aminopropane1,2-diol; Alanine; 6-Phosphogluconate; Ophthalmate	4-Amino-3-hydroxybutyrate; 2-Aminoethylphosphonate; UDP-glucose/UDP-galactose; N^5^-Ethylglutamine; UDP-glucuronate; 2-Hydroxybutyrate; Octanoate; Gly leu
PiaMarlene Nissen (2011) [46]	12	12	Piglets with low birth weight	Umbilical cord plasma	110 d of gestation	GC-MS	Untargeted	Myoinositol; D-chiro-inositol	None
Qien Wang (2020) [47]	53	57	Fetal rats of low protein diet maternal rats	Fetal kidney	20 d of gestation	GC-MS LC-MS	Untargeted	Xanthine; Xanthosine; L-lysine; Oleate; Arachidonate; Phenylalanine; Glutamine; Guanine	N-acetylornithine; Arginine; GMP; UMP; Acetyl-CoA; Carbamoyl phosphate; dAMP; UDP; TMP; dTDP; Urea; Pantothenate

**Table 6:** High Frequency Metabolic Biomarkers of Animal Studies.

No	Metabolites	Hits	Up	Down
1	Arginine	4	-	Fetal rats plasma [40] Fetal sheep skeletal muscle+ arterial and venous plasma [41] Piglets umbilical vein plasma [43] Fetal rats kidney [47]
2	Glutamine	3	Calves muscle [44] Fetal rats kidney [47]	Piglets umbilical vein plasma [43]
3	Phenylalanine	3	Calves muscle [44] Fetal rats kidney [47]	Piglets umbilical vein plasma [43]
4	Proline	3	Calves muscle [44]	Fetal rats plasma [40] Piglets umbilical vein plasma [43]

## Supplementary Materials

The following supporting information can be downloaded at: https://www.mdpi.com/article/10.3390/metabo12090860/s1, Table S1: Results of the Pathway Analysis of Neonatal Studies; Table S2: Results of the Pathway Analysis of Maternal Studies.
