# Supplementary material for: The Exploration of Fetal Growth Restriction Based on Metabolomics: A Systematic Review
Source: Metabolites. 2022 Sep 13;12(9):860. doi: 10.3390/metabo12090860 (PMC9501562; doi:10.3390/metabo12090860)
Supplement: Supplementary file 1 [file metabolites-12-00860-s001.zip › supplementary files.pdf]

Table S1 Results of the Pathway Analysis of Human Neonatal Studies

Pathway name	Total	Hits	Raw p	Holm adjust	FDR	Impact
Aminoacyl-tRNA biosynthesis	48	17	0.00000	0.00000	5.15E-14	0.00000
Valine, leucine and isoleucine biosynthesis	8	4	0.00004	0.00332	0.0016782	0.00000
Glyoxylate and dicarboxylate metabolism	32	6	0.00022	0.01795	0.0061293	0.13757
Arginine biosynthesis	14	4	0.00050	0.04071	0.0098223	0.42132
Arginine and proline metabolism	38	6	0.00058	0.04677	0.0098223	0.29477
Alanine, aspartate and glutamate metabolism	28	5	0.00098	0.07759	0.01375	0.31090
Phenylalanine, tyrosine and tryptophan biosynthesis	4	2	0.00477	0.37180	0.0572	1.00000
Synthesis and degradation of ketone bodies	5	2	0.00780	0.60043	0.076095	0.60000
Butanoate metabolism	15	3	0.00815	0.61963	0.076095	0.11111
Nitrogen metabolism	6	2	0.01148	0.86110	0.087676	0.00000
D-Glutamine and D-glutamate metabolism	6	2	0.01148	0.86110	0.087676	0.50000
Glycine, serine and threonine metabolism	33	4	0.01372	1.00000	0.096009	0.24577
Valine, leucine and isoleucine degradation	40	4	0.02642	1.00000	0.17073	0.00000
Tyrosine metabolism	42	4	0.03103	1.00000	0.17913	0.38029
Phenylalanine metabolism	10	2	0.03199	1.00000	0.17913	0.35714
Glutathione metabolism	28	3	0.04514	1.00000	0.23696	0.10839

Table S2 Results of the Pathway Analysis of Human Maternal Studies

Pathway name	Total	Hits	Raw p	Holm adjust	FDR	Impact
Aminoacyl-tRNA biosynthesis	48	16	0.00000	0.00000	0.00000	0.00000
Valine, leucine and isoleucine biosynthesis	8	6	0.00000	0.00004	0.00002	0.00000
Glutathione metabolism	28	7	0.00040	0.03267	0.00897	0.38582
Arginine biosynthesis	14	5	0.00048	0.03853	0.00897	0.25380
Arginine and proline metabolism	38	8	0.00053	0.04271	0.00897	0.53597
Glyoxylate and dicarboxylate metabolism	32	7	0.00095	0.07543	0.01337	0.13757
Alanine, aspartate and glutamate metabolism	28	6	0.00253	0.19697	0.03030	0.53446
Glycine, serine and threonine metabolism	33	6	0.00602	0.46326	0.06317	0.33870
Glycerophospholipid metabolism	36	6	0.00933	0.70904	0.08708	0.29964
Phenylalanine, tyrosine and tryptophan biosynthesis	4	2	0.01511	1.00000	0.12695	1.00000
Synthesis and degradation of ketone bodies	5	2	0.02434	1.00000	0.18585	0.60000
D-Glutamine and D-glutamate metabolism	6	2	0.03528	1.00000	0.22796	0.50000
Nitrogen metabolism	6	2	0.03528	1.00000	0.22796	0.00000
Butanoate metabolism	15	3	0.03969	1.00000	0.23812	0.11111
Glycolysis / Gluconeogenesis	26	4	0.04299	1.00000	0.24073	0.12971
Histidine metabolism	16	3	0.04705	1.00000	0.24700	0.09016
